# Melatonin Treatment Maintains the Quality of Fresh-Cut *Gastrodia elata* under Low-Temperature Conditions by Regulating Reactive Oxygen Species Metabolism and Phenylpropanoid Pathway

**DOI:** 10.3390/ijms241814284

**Published:** 2023-09-19

**Authors:** Boyu Dong, Fangfang Da, Yulong Chen, Xiaochun Ding

**Affiliations:** 1 School of Chinese Ethnic Medicine, Guizhou Minzu University, Guiyang 550025, China; dafangfang@gzmu.edu.cn (F.D.); 15772710928@163.com (Y.C.); 2 Key Laboratory of Guizhou Ethnic Medicine Resource Development and Utilization in Guizhou Minzu University, State Ethnic Affairs Commission, Guiyang 550025, China; 3 Engineering Research Center for Fruit Crops of Guizhou Province, Key Laboratory of Plant Resource Conservation and Germplasm Innovation in Mountainous Region (Ministry of Education), College of Agriculture, Guizhou University, Guiyang 550025, China

**Keywords:** fresh-cut *Gastrodia elata*, melatonin, ROS metabolism, phenylpropanoid pathway

## Abstract

The application of melatonin (MT) has been shown to improve the quality during the storage of fruits and vegetables. The primary objective of this study is to investigate the effects of MT on the quality of fresh-cut *Gastrodia elata* during low-temperature (4 °C) storage. The results indicated that MT treatment not only suppressed the respiratory rate and malondialdehyde content but also slowed down the decline in total acidity and total soluble solids, effectively inhibiting microbial growth and enhancing the product safety of fresh-cut *G. elata*. The treatment with MT reduced the superoxide anions and hydrogen peroxide production, as well as inhibiting the activity and expression of peroxidase and polyphenol oxidase. Additionally, it led to increased activity and the expression of antioxidant-related enzymes, including superoxide dismutase, catalase, ascorbate peroxidase, glutathione reductase, monodehydroascorbate reductase, and dehydroascorbate reductase, while also resulting in elevated levels of ascorbic acid and glutathione. Furthermore, the treatment with MT induced an increase in the total phenolic and flavonoid content of fresh-cut *G. elata* and enhanced the activity and expression of key enzymes involved in the phenylpropanoid pathway (phenylalanine ammonia-lyase, cinnamate-4-hydroxylase, 4-coumarate: CoA ligase). In summary, MT enhances the antioxidant capacity by activating both the ROS metabolism and phenylpropanoid pathway, thus maintaining the quality of fresh-cut *G. elata*.

## 1. Introduction

*Gastrodia elata*, belonging to the Orchidaceae family, is rich in functional components, such as gastrodin, amino acids, and polysaccharides [1]. In 2019, *G. elata* was designated as a pilot variety for the homology of medicine and food by the Chinese health commission. With *G. elata* being recognized as a food with medicinal properties, its applications as a food with medicinal properties are rapidly expanding [2]. Storing fresh *G. elata* for extended periods is challenging, and it is prone to rot [3]. Currently, research on the storage and preservation of fresh *G. elata* is relatively limited.

Melatonin (MT), a derivative of the essential amino acid tryptophan, is recognized as a novel plant hormone due to its diverse functions [4]. Prior research has shown that MT can scavenge reactive oxygen species (ROS) and enhance the quality of fruits and vegetables. Postharvest MT treatment has been shown to maintain the quality and delay senescence in jujube [5], plum [6], apple [7], and eggplant [8] during storage. However, the impact of MT treatment on the quality of fresh-cut *G. elata* remains unknown.

It has been confirmed that ROS can directly interact with pathogens or act as signaling molecules in plant defense responses. However, excessive ROS can damage cells, disrupt cell membrane integrity, and accelerate plant aging [9]. Plants regulate ROS metabolism-related enzymes, such as superoxide dismutase (SOD), catalase (CAT), ascorbate peroxidase (APX), and glutathione reductase (GR), along with antioxidant substances, like ascorbic acid (AsA) and glutathione (GSH), to maintain the balance of ROS [10]. Previous studies have proven that postharvest inducer treatments could enhance the enzymatic activity of oxygen metabolism-related enzymes, thereby improving the antioxidant capacity of apples [11], ginger rhizomes [12], pears [13], and blueberries [14].

The phenylpropanoid pathway, an integral secondary metabolic route within plants, assumes a pivotal responsibility in bolstering plant antioxidant mechanisms and fortifying resilience against diseases. A plethora of indispensable antimicrobial agents present in plants, encompassing flavonoids and phenolic compounds, originate from the intricate progression of the phenylpropanoid pathway, either through direct or indirect biosynthetic routes [15]. Pre-treatment with hot air has been shown to enhance the biosynthesis of phenolic compounds and maintain high antioxidant activity in fresh-cut pitaya fruits [16]. Furthermore, previous studies have demonstrated that the accumulation of the phenylpropanoid pathway can enhance the antioxidant capacity of fresh-cut Agaricus bisporus [17], fresh-cut pitaya [18,19], and fresh-cut potato strips [20]. However, the impact of MT on the antioxidant capacity and phenylpropanoid pathway in fresh-cut *G. elata* has not been investigated yet.

The primary objective of the present study was to examine the impacts of MT treatment on the following: (1) the quality of fresh-cut *G. elata* during low-temperature (4 °C) storage, and (2) the metabolism of antioxidant and phenylpropanoid of fresh-cut *G. elata*, that is, to assess the efficacy of MT in improving the overall quality of fresh-cut *G. elata*.

## 2. Results

### 2.1. Effect of MT on Phenotypic Observation, Weight Loss, Respiratory Rate, Total Soluble Solid (TSS), Titratable Acid (TA), and Malondialdehyde (MDA) in Fresh-Cut G. elata

**Figure 1:**
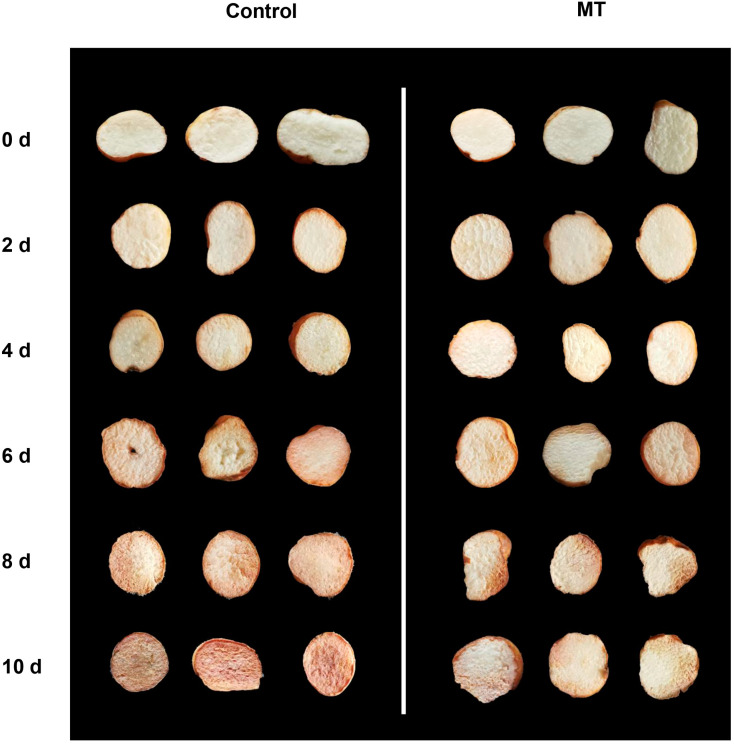
Visual appearance of fresh-cut *G. elata* after 0 and 50 μmol/L melatonin treatment during storage at 4 °C.

**Figure 2:**
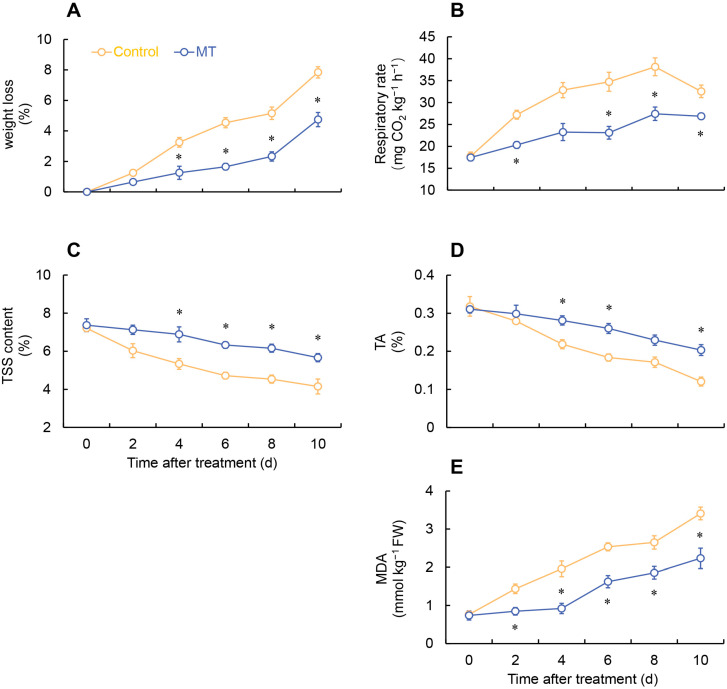
Effect of melatonin treatment on weight loss (**A**), respiratory rate (**B**), TSS (**C**), TA (**D**), and MDA (**E**) at 4 °C. * denotes significant difference at the level of *p* < 0.05. Vertical bars represent the standard errors of the means (±SE).

As depicted in Figure 1, the control group of fresh-cut *G. elata* exhibited significant browning on the surface after 6 days of storage at 4 °C. However, MT treatment effectively inhibited the degree of browning in fresh-cut *G. elata* during the same period (Figure 1). In both groups, the fresh-cut *G. elata* exhibited an increasing trend in weight loss throughout the storage duration. However, the rate of weight loss from days 4 to 10 was notably lower in the MT-treated group compared to the control group (Figure 2A). After MT treatment, the respiratory rate of fresh-cut *G. elata* remained consistently lower than the control throughout the storage stage and substantially lower at 2 and 6–10 days compared to the control (Figure 2B). Both the MT treatment and control groups of fresh-cut *G. elata* exhibited a decreasing trend in TSS and TA throughout the entire experimental stage. However, the MT treatment significantly delayed the decrease in TSS and TA of fresh-cut *G. elata* from days 4 to 10 and 4 to 6 and 10 (Figure 2C,D). The MDA content showed an increasing trend in both the control and MT treatment groups. However, the MT treatment markedly reduced the MDA content throughout the entire storage period (Figure 2E).

### 2.2. Effect of Melatonin Treatment on Microbial Colony Counts of Fresh-Cut G. elata

**Figure 3:**
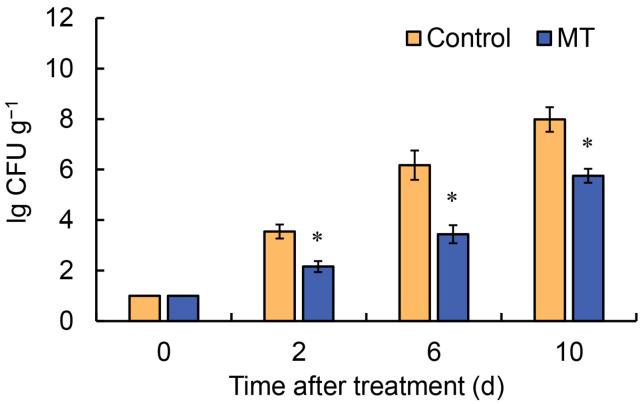
Microbial colony counts in fresh-cut *G. elata* during storage at 4 °C after treatment with melatonin or water (control). Vertical bars represent standard error of the means of quadruplicate assays. Asterisks represent values that are significantly different between control and melatonin-treated fruit at the same time point (* *p* < 0.05).

As *G. elata* tissue integrity is compromised, it becomes highly susceptible to microbial invasion and growth. To ensure the safety of food products containing *G. elata* for consumption, effective control measures are essential. At 2, 6, and 10 d, the microbial colony counts in untreated *G. elata* (control) were 3.5 × 10^3^, 1.5 × 10^6^, and 9.6 × 10^7^ CFU g^−1^, respectively (Figure 3). However, treatment with MT effectively inhibited microbial proliferation, resulting in colony counts of 1.55 × 10^2^, 2.8 × 10^4^, and 6.6 × 10^5^ CFU g^−1^ at 2, 6, and 10 d, respectively (Figure 3). Thus, the application of MT effectively inhibited microbial colony counts in *G. elata* samples over a 10-day period.

### 2.3. Effect of MT on ROS Metabolism

**Figure 4:**
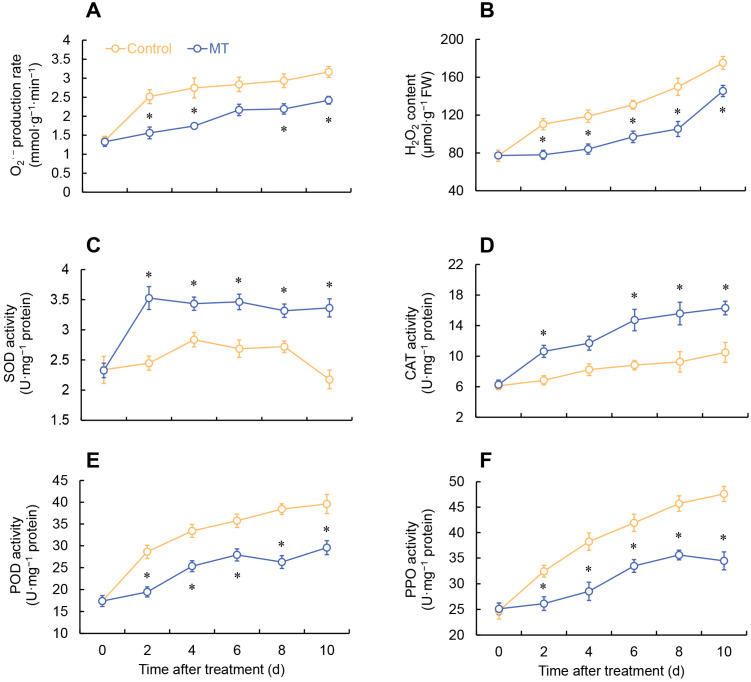
Changes in O_2_^•−^ production rate (**A**), H_2_O_2_ content (**B**), and the activity of SOD (**C**), CAT (**D**), POD (**E**), and PPO (**F**) in fresh-cut *G. elata* after melatonin treatment during storage at 4 °C. * denotes significant difference at the level of *p* < 0.05. Vertical bars represent the standard errors of the means (±SE).

As shown in Figure 4A,B, both the superoxide anion (O_2_^•−^) production rate and hydrogen peroxide (H_2_O_2_) content in both MT treatment and control exhibited a gradual increasing trend. Specifically, the group treated with MT exhibited a notable reduction in the production rate of O_2_^•−^ compared to the control group on days 2 and 5–7 (Figure 4A). Additionally, throughout the entire experimental period, the MT-treated group consistently demonstrated significantly lower levels of H_2_O_2_ content compared to the control group (Figure 4B). The MT-treated group exhibited a rapid increase in SOD activity within the first 2 days, reaching its peak on day 2. Although there were fluctuations in SOD activity afterward, it consistently remained significantly higher than the control group (Figure 4C). During the storage period, both the MT-treated and control groups demonstrated a progressive increase in CAT activity. Notably, the MT-treated group exhibited significantly higher CAT activity than the control group on days 2 and 6–10 (Figure 4D). The control group displayed an upward trend in peroxidase (POD) and polyphenol oxidase (PPO) activities. In contrast, the MT-treated group exhibited significantly suppressed POD and PPO activities compared to the control group (Figure 4E,F).

**Figure 5:**
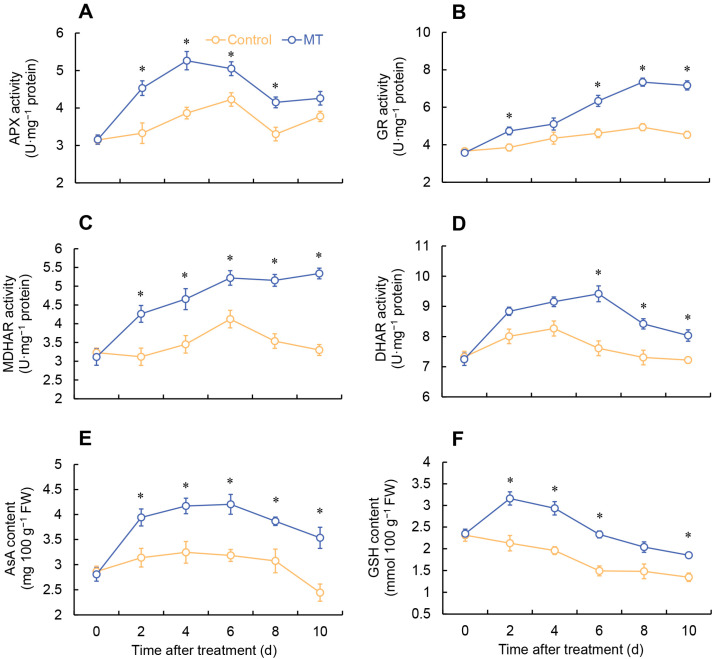
Changes in the activity of APX (**A**), GR (**B**), MDHAR (**C**), and DHAR (**D**), and the content of AsA (**E**) and GSH (**F**) in fresh-cut *G. elata* after melatonin treatment during storage at 4 °C. * denotes significant difference at the level of *p* < 0.05. Vertical bars represent the standard errors of the means (±SE).

In the MT treatment, APX activity rapidly increased from 0 to 4 days, followed by a decrease from 4 to 8 days. Specifically, the MT-treated group showed considerably higher APX activity compared to the control on days 2–8. On the other hand, the control exhibited an increase in APX activity from 0 to 6 days, followed by fluctuations (Figure 5A). The GR activity in the MT treatment group was elevated from 0 to 8 days, followed by a decrease. Throughout the entire stage, the MT-treated group consistently exhibited greater GR activity compared to the control, with remarkable differences observed on days 2 and 6–10 (Figure 5B). The monodehydroascorbate reductase (MDHAR) activity rose from 0 to 6 days in the MT-treated group, followed by fluctuations. Throughout the entire storage period, the MT treatment consistently exhibited significantly higher MDHAR activity (Figure 5C). The control group showed a progressive increase in dehydroascorbate reductase (DHAR) activity from the initial day to day 6, followed by a subsequent decline. In contrast, the MT-treated group demonstrated a noteworthy rise in DHAR activity, notably surpassing the control on days 6 to 10 (Figure 5D).

### 2.4. Determination of AsA and GSH Content

Both the MT-treated and control groups showed a similar trend in terms of the change in AsA content, reaching their respective peak values at 6 and 4 days, followed by a decrease. Throughout the entire storage duration, the MT treatment consistently showcased notably elevated levels of AsA content in comparison to the control group, as illustrated in Figure 5E. The GSH content consistently reduced in the control. In contrast, the MT-treated group exhibited a continuous increase in GSH content from 0 to 2 days, followed by a decrease. Additionally, during days 2–6 and 10, the MT-treated group demonstrated notably higher GSH content compared to the control (Figure 5F).

### 2.5. Effects of MT Treatment on Phenylpropanoid Pathway 

**Figure 6:**
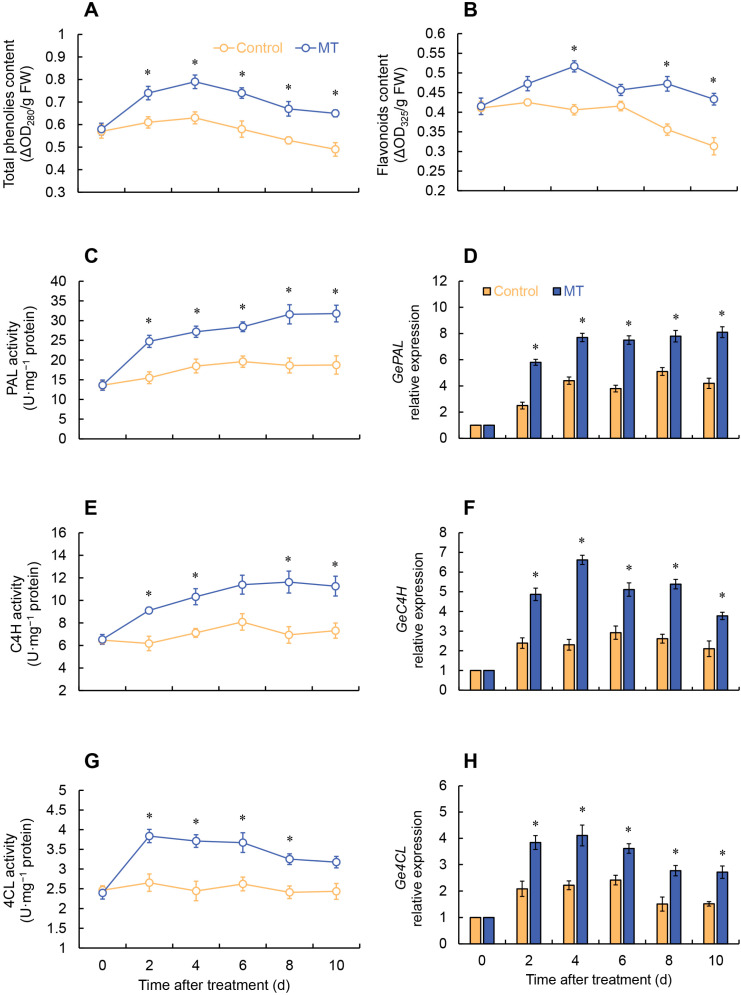
Changes in the content of total phenols (**A**) and flavonoids (**B**), and the activity and expression of PAL (**C**,**D**), C4H (**E**,**F**), and 4CL (**G**,**H**) in fresh-cut *G. elata* after melatonin treatment during storage at 4 °C. * denotes significant difference at the level of *p* < 0.05. Vertical bars represent the standard errors of the means (±SE).

The total phenolic content in both the control and MT treatment groups increased from 0 to 4 days and then declined. Nonetheless, over the entirety of the storage interval, the MT treatment consistently demonstrated significantly augmented levels when juxtaposed with the control group, as depicted in Figure 6A. The flavonoid content in the control exhibited fluctuations from 0 to 6 days, followed by a decrease. In contrast, the MT-treated group showed an increase in flavonoid content from 0 to 4 days, reaching its maximum level. Additionally, the MT-treated group exhibited an essential increase on days 4 and 8–10 (Figure 6B). The phenylalanine ammonia-lyase (PAL) activity of MT treatment increased consistently throughout the entire storage period and was considerably greater than the control. In contrast, the PAL activity in the control increased from 0 to 6 days and then fluctuated thereafter (Figure 6C). After MT treatment, the cinnamate-4-hydroxylase (C4H) activity of fresh-cut *G. elata* was markedly higher than the control at 2–4 and 8–10 days (Figure 6E). The control showed no significant changes in 4-coumarate: CoA ligase (4CL) activity throughout the entire experimental stage. However, the MT treatment exhibited a rapid increase in 4CL activity from 0 to 2 days, reaching its peak, followed by a gradual decline, and it was substantially greater than the control group from 2 to 8 days (Figure 6G). As shown in Figure 6, MT treatment markedly elevated the expression of key enzymes involved in phenylpropane metabolism (PAL, C4H, 4CL) throughout the entire storage stage (Figure 6D,F,H).

### 2.6. Gene Expressions of ROS Metabolism of Fresh-Cut G. elata

**Figure 7:**
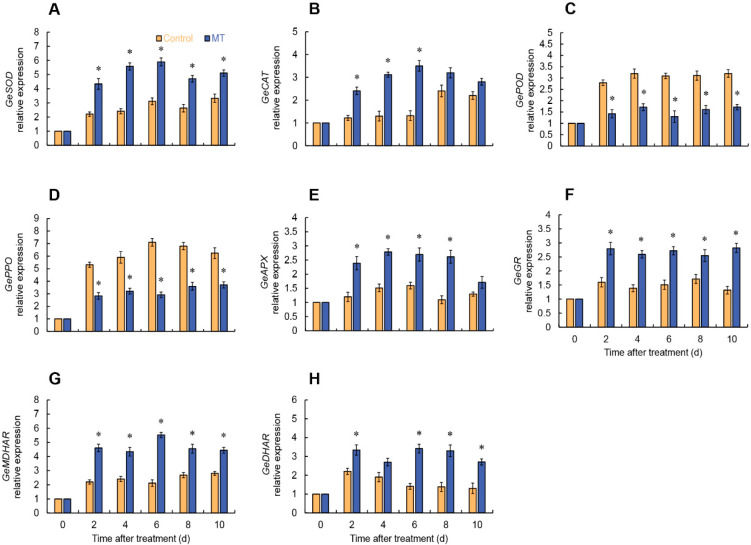
Expression of *GeSOD* (**A**), *GeCAT* (**B**), *GePOD* (**C**), *GePPO* (**D**), *GeAPX* (**E**), *GeGR* (**F**), *GeMDHAR* (**G**), and *GeDHAR* (**H**) in fresh-cut *G. elata* after melatonin treatment during storage at 4 °C. * denotes significant difference at the level of *p* < 0.05. Vertical bars represent the standard errors of the means (±SE).

In the MT treatment, *GeSOD* and *GeCAT* expressions rapidly increased from 2 days and reached their peak at 6 days. In particular, the *GeSOD* expression in the MT treatment remained substantially higher than the control throughout the storage period (Figure 7A), while the *GeCAT* expression was markedly higher than the control from 2 to 6 days (Figure 7B). After MT treatment, *GePOD* and *GePPO* expressions in fresh-cut *G. elata* were considerably lower compared to the control throughout the experimental stage (Figure 7C,D). According to Figure 6, it is evident that MT treatment considerably enhanced the expression levels of key enzymes involved in the ascorbate–glutathione cycle (AsA-GSH) (APX, GR, MDHAR, DHAR) to varying degrees (Figure 7E–H).

## 3. Discussion

After plants are wounded, the respiration and transpiration rates accelerate, which is an important factor leading to the aging and quality deterioration of fresh-cut fruits [21]. Slowing down respiration and transpiration is an effective approach to preserve the quality of fresh-cut fruits and vegetables. Melatonin is a vital hormone in the human body that plays a crucial role in regulating the biological clock and sleep cycles [2]. However, its presence in plants is typically in trace amounts, serving as a natural plant signaling molecule, and generally does not have adverse effects on human health [22]. This study found that MT treatment effectively reduces the respiration rate of fresh-cut *G. elata*, delays quality deterioration, and significantly decreases the MDA content during storage, thereby slowing down the process of cell membrane lipid peroxidation. Tan et al. found that MT treatment effectively reduces the respiration rate of Chinese flowering cabbage, delaying the increase in weight loss, thus ensuring its postharvest quality [23]. In addition, in a study on wax apple, it was found that postharvest MT treatment significantly delayed the increase in weight loss and effectively reduced MDA content, alleviating cell membrane damage in wax apple [24].

During postharvest respiration, both TA and TSS are utilized as substrates, and TA and TSS are important indicators of plant quality after harvest. However, an excessive respiration rate accelerates the depletion of TSS and TA, leading to a decline in plant quality [25]. This study found that MT effectively reduces the depletion of TSS and TA in fresh-cut *G. elata*, which is consistent with the significant decrease in the respiration rate observed after treatment. Likewise, similar results were obtained in studies on Chinese flowering cabbage [23] and wax apple [24] with MT treatment after harvest. As *G. elata* tissue integrity is compromised, it becomes highly susceptible to microbial invasion and growth. To ensure the safety of *G. elata*, microbial colony counts were measured. The results showed that the application of MT effectively inhibited microbial colony counts in *G. elata* samples over a 10-day period. Furthermore, these findings are consistent with numerous previous studies, reporting that the application of MT can induce postharvest disease resistance in various fruits and vegetables [22].

When plants encounter abiotic stress, they produce a notable quantity of ROS. Moderate levels of ROS can function as signaling molecules that trigger the defense system in plants, whereas elevated concentrations of ROS can lead to oxidative damage in plants [26]. In response to this situation, plants possess a comprehensive antioxidant system, comprising antioxidant enzymes, like SOD and CAT, along with the AsA-GSH cycle. These mechanisms play a vital role in neutralizing excessive ROS and preserving a harmonious ROS equilibrium [9]. This study found that MT treatment effectively enhances the activities and expressions of SOD and CAT in fresh-cut *G. elata*. Simultaneously, it significantly reduces the rate of O_2_^•−^ generation and the content of H_2_O_2_ during storage. Prior studies have found that post-treatment with MT significantly reduces H_2_O_2_ levels and enhances the activities of SOD and CAT in ‘Newhall’ navel oranges. This effectively delays the postharvest senescence of the oranges and maintains their quality [4]. According to Chen et al., the application of MT in wax apples has been shown to effectively decrease ROS accumulation while simultaneously enhancing the activities of antioxidant enzymes, like SOD and CAT. These findings suggest that the external application of MT mitigates postharvest decay in wax apples by maintaining a delicate equilibrium between ROS generation and the activity of antioxidant system [24]. Furthermore, postharvest treatment with MT has been demonstrated to delay the senescence of various fruits and vegetables and enhance their disease resistance by regulating the ROS metabolism and antioxidant capacity. This includes fresh-cut broccoli [27], sweet cherries [28], and mango fruit [29]. Therefore, we hypothesize that MT reduces the excessive ROS levels in fresh-cut *G. elata* by activating SOD and CAT.

In plants, the AsA-GSH cycle serves as an essential ROS clearance system, where key enzymes, such as APX, GR, and glutathione peroxidase, play critical roles. These enzymes participate directly or indirectly in the AsA-GSH cycle, protecting cells from oxidative damage [30]. The function of this cycle is to neutralize ROS by converting AsA and GSH to their oxidized forms, thereby maintaining the redox balance within the cell. APX converts AsA to dehydroascorbate [31], while GR reduces dehydroascorbate back to AsA and converts oxidized glutathione to reduced glutathione [32]. The synergistic action of these enzymes helps maintain the redox balance within plant cells, protecting them from oxidative damage. This study discovered that the application of MT treatment significantly boosts the activity and expression of APX and GR in fresh-cut *G. elata*. According to research conducted by Aghdam et al., the application of MT treatment has been found to effectively boost the antioxidant capacity of postharvest pomegranate. This positive effect is attributed to the significant increase in the activities of two important antioxidant enzymes, APX and GR, in the pomegranate fruit [33]. Furthermore, similar results have been observed in studies on sweet cherries [28], jujubes [5], and white mushroom [34] with MT treatment.

The AsA-GSH cycle is a crucial system for maintaining cellular redox homeostasis, with AsA and GSH serving as major non-enzymatic antioxidants. AsA exhibits potent antioxidant properties and can be recycled back to its reduced form (AsA) from its oxidized form (dehydroascorbic acid) through interactions with DHAR and GSH [35]. Additionally, MDHAR is also involved in the AsA-GSH cycle, facilitating the conversion of monodehydroascorbate to AsA and, thereby, contributing to the normal functioning of this cycle [27]. During this investigation, it was observed that MT treatment could enhance the activities and expressions of MDHAR and DHAR to varying degrees, effectively increasing the contents of AsA and GSH during storage. Previous studies have revealed that exogenous MT can enhance the AsA-GSH cycle by increasing AsA content, thereby maintaining the quality of guava fruit and enhancing its disease resistance [36]. Treatment with MT has been shown to increase the AsA content in papaya fruit and improve its quality [37]. Aghdam et al. demonstrated that the application of MT effectively improved the levels of AsA and GSH in pomegranate fruit [33]. This resulted in improved antioxidant capacity and the preservation of fruit quality. Furthermore, Wang et al. demonstrated that exogenous MT treatment enhanced the activities of AsA-GSH cycle-related enzymes, MDHAR and DHAR, during the storage period of sweet cherries. It also increased the contents of AsA and GSH, thereby maintaining the postharvest quality of sweet cherries [28]. The aforementioned results suggest that MT treatment could activate the activity and expression of AsA-GSH cycle-related enzymes, such as APX, GR, MDHAR, and DHAR, further increasing the levels of AsA and GSH. Consequently, this helps eliminate excessive ROS in fresh-cut *G. elata* and maintain ROS balance.

When fresh-cut fruits and vegetables are subjected to cutting or other forms of mechanical stress, they respond to this injury by synthesizing higher levels of phenolic compounds, thereby enhancing their antioxidant capacity [38]. These phenolic compounds include flavonoids, anthocyanins, and phenolic acids, which exhibit strong antioxidant properties and can neutralize ROS, thereby reducing cellular oxidative damage [16]. Through this mechanism, fresh-cut fruits and vegetables can rapidly respond to cutting, enhance their antioxidant defense, prolong shelf life, and maintain nutritional value and texture. Based on this research, the application of MT treatment was observed to significantly boost the accumulation of total phenolics and flavonoids in fresh-cut *G. elata* during storage, thus improving its antioxidant capacity. A previous experiment demonstrated that exogenous MT treatment could enhance the total phenolic content in guava fruit, thereby maintaining its antioxidant capacity and enhancing its disease resistance [36]. MT treatment has also been shown to enhance the accumulation of total phenolics in sweet cherry fruit, thereby enhancing fruit antioxidant capacity and maintaining storage quality [39]. Furthermore, in studies conducted on cherry tomato fruit [40] and pomegranate fruits [33], it has been found that MT treatment could increase the content of phenolic compounds.

The phenylpropanoid pathway in plants is an important secondary metabolic pathway involved in the synthesis of various phenolic compounds. Within this pathway, PAL, 4CL, and C4H are three key rate-limiting enzymes [15]. PAL functions as the primary regulatory enzyme in phenylpropanoid metabolism, converting L-phenylalanine into cinnamic acid. Subsequently, C4H and 4CL catalyze the hydroxylation of different types of hydroxycinnamic acids into their corresponding thioesters, which then enter the synthesis pathway of phenolic compounds, flavonoids, and anthocyanins [41]. In this research, it was observed that the application of MT treatment led to notable increases in both the activities and expressions of PAL, C4H, and 4CL. These enhancements indirectly resulted in elevated levels of phenolic compounds. Previous research has revealed that MT treatment of blueberry fruits leads to increased PAL, C4H, and 4CL activities and expressions, ensuring enhanced antioxidant capacity and disease resistance [22]. Similar findings in litchi fruit demonstrate that MT treatment is associated with the maintenance of fruit quality and the activities of PAL, C4H, and 4CL [42]. Additionally, similar conclusions have been obtained in studies conducted on cherry tomato [39], tomato fruits [43], and pomegranate fruits [33]. However, at the same time, certain phenolic compounds, such as caffeic acid and catechins, are susceptible to oxidation by PPO and POD, resulting in the formation of quinone polymers and brown pigments, leading to rapid browning of fresh-cut fruits and vegetables [18]. This phenomenon is attributed to the oxidation of the catechol structure present in the phenolic compounds, which generates quinone structures that subsequently polymerize to form oligomers and brown pigments. Our research results indicate that MT treatment effectively inhibits the activities and expressions of POD and PPO, leading to a significant reduction in the browning extent of fresh-cut *G. elata* during storage, which is consistent with the results depicted in Figure 1. Li et al. conducted research on fresh-cut white pitaya fruit and reported similar results. They found that subjecting the fruit to hot-air pre-treatment resulted in enhanced activities of PAL, C4H, and 4CL, while simultaneously inhibiting the activities of POD and PPO [16]. This treatment effectively prevented reductions in total phenolic content and successfully controlled browning in fresh-cut white pitaya fruit. Therefore, we hypothesize that MT treatment could enhance the antioxidant capacity of fresh-cut *G. elata* by activating the rapid accumulation of total phenolics through the phenylpropanoid pathway. Simultaneously, it effectively inhibited the activities of POD and PPO, preventing premature browning in fresh-cut *G. elata* and, thus, maintaining its postharvest quality.

## 4. Materials and Methods

### 4.1. Fruit and Treatment

The *G. elata* was harvested from Zhaotong (103°79′ E, 27°38′ N), Yunnan Province, China, and taken to the laboratory by car. *G. elata* that was free from diseases, pests, and mechanical damage and of uniform size was selected, sterilized with 0.2% (*v*/*v*) sodium hypochlorite solution for 2 min, then rinsed with water and randomly divided into two treatment groups of 90 *G. elata* each. The two sets of slices were manually peeled and cut into segments (each *G. elata* yielding 6 slices), with each slice being approximately 1 cm thick and 3–3.5 cm in diameter on an ultra-clean bench. In the first group, the *G. elata* slices were uniformly immersed in 50 μM MT for 5 min. Our initial experiments involved dissolving melatonin (MT) in ethanol at a final concentration of 0.01% and then diluting it with water to create concentrations of 25, 50, 100, and 200 μM. Preliminary results indicated that a concentration of 50 μM MT demonstrated the most effective inhibition of browning (data not presented). Consequently, we selected the 50 μM concentration for our subsequent analysis in this study. The control group slices were immersed in a solution of 0.01% ethanol with hyperpure water. Subsequently, all the slices were carefully sealed and packed into modified atmosphere polypropylene containers (dimensions: 180 mm × 135 mm × 8 mm) and stored under conditions of 4 °C and 80–85% relative humidity. At 0, 2, 4, 6, 8, and 10 days of 4 °C storage, phenotypic observations, physiological, and biochemical indexes were performed, and fresh-cut *G. elata* was snap-frozen with liquefied nitrogen and stored in an ultra-low-temperature refrigerator (−80 °C) for subsequent experiments. Three biological replicates were used for all index determinations.

### 4.2. Determination of Weight Loss, Respiratory Rate, TSS, TA, and MDA

The weight loss rate (%) is calculated as: [(initial fresh-cut *G. elata* weight − present fresh-cut *G. elata* weight)]/initial fresh-cut *G. elata* weight] × 100. The respiration rate was assessed using a dual-wavelength infrared carbon dioxide analyzer (HM-GX20, Shandong Hengmei Electronic Technology Co., Ltd., Weifang, China). Changes in CO_2_ concentration were tracked by enclosing four slices of fresh-cut *G. elata* in a sealed 100 mL container linked to the analyzer, forming a closed gas circulation system. Measurements were recorded at 1 min intervals over five consecutive instances, and the respiration rates were expressed as mg CO_2_ kg^−1^h^−1^ fresh weight. TSS was measured using an ATAGO-PAL-1 digital refractometer, and TA was titrated using NaOH (pH 13.0, 0.1 mol L^−1^) and then expressed as a percentage relative to TSS and TA. MDA content was determined using the method of Gao et al., where absorbance was measured at 450, 532, and 600 nm and expressed as mmol kg^−1^ FW [44].

### 4.3. Assays for ROS Production and the Activity of Antioxidant Scavenging Enzymes

O_2_^•−^ production rate and H_2_O_2_ were measured on a UV spectrophotometer using O_2_^•−^ and H_2_O_2_ Quantification Kits (Beyotime Biotechnology Co., Ltd., Beijing, China), respectively, in accordance with the specific procedures suggested by the producer. The rate of O_2_^•−^ generation and H_2_O_2_ content was indicated in mol kg^−1^ min^−1^ and mmol kg^−1^ FW, respectively.

For assessment, the activity of antioxidant scavenging enzyme, SOD, APX, CAT, and GR extraction procedures are detailed in Ding et al. [45].

DHAR and MDHAR and were extracted according to the instructions of the kit (Solarbio Life Sciences. Co., Ltd., Beijing, China).

SOD activity was established by assessing the capacity to suppress the reductive effect of nitro blue tetrazolium in the presence of light [45]. 1U represents the amount of enzyme required to depress 50% of the photo-reduction reaction of NBT.

CAT activity was defined by examining the change in absorbance caused by H_2_O_2_ content at 240 nm [14]. 1U of CAT activity was defined as the amount of enzyme per minute that caused the decomposition of 1 μmol of H_2_O_2_. 

APX activity was evaluated through the changed absorbance (290 nm) caused by the oxygenation of AsA after the addition of H_2_O_2_ [14]. 1U of APX activity was the amount of enzyme per minute that caused the oxidation of 1 μmol of AsA. 

GR activity was measured as the change in absorbance due to NADPH oxidation in the absence of oxidized glutathione [14]. GR activity of 1U was calculated as the enzyme amount per minute leading to 1 nmol of NADPH oxidation. 

The activities of DHAR and MDHAR were identified according to the kit instructions. 1U of the activity of DHAR was defined as the enzyme amount catalyzing the production of 1 μmol of AsA per second, and MDHAR was determined as the enzyme amount per second catalyzing 1 μmol NADPH oxidation. All the above antioxidant scavenging enzyme activities were shown in U kg^−1^ FW.

### 4.4. Activities of POD and PPO

In this study, we conducted the extraction of frozen tissues weighing 2.0 g using a 50 mM pre-chilled phosphate buffer solution (PBS) with a pH of 7.0, supplemented with 1% polyvinylpyrrolidone (PVP). The resultant supernatants served as the enzymatic source for the assessment of POD and PPO activities. To measure POD (EC 1.11.1.7) activity, the method outlined by Li et al. was adopted [16]. The reaction system consisted of a 1 mL mixture containing 50 mM PBS (pH 7.0), 20 mM H_2_O_2_, 1% guaiacol, and 50 μL of the extracted supernatant. The monitoring of absorbance changes at 560 nm allowed us to determine the enzymatic activity, with one unit of POD defined as the catalysis of 1 μmol of guaiacol oxidation per minute. For the assessment of PPO (EC 1.10.3.1) activity, we prepared a reaction mixture of 1 mL comprising 50 mM catechol, 50 μL of the extracted supernatant, and 0.1 M PBS at pH 6.8. The observation of absorbance changes at 420 nm facilitated the determination of enzymatic activity, with one unit of PPO defined as the oxidation of 1 nmol of catechol per minute. All the above enzyme activities were shown in 10^6^ U kg^−1^ protein.

### 4.5. Determination of AsA and GSH Content

The contents of AsA and GSH content were determined with reference to Dong et al. and expressed as mmol kg^−1^ FW, g kg^−1^ FW, mg kg^−1^ FW, and mmol kg^−1^ FW, respectively [46].

### 4.6. Analysis of the Metabolite Content in the Phenylpropanoid Pathway

Total phenolic, flavonoid, and lignin contents were extracted and measured using the method described by Liu et al. Total phenolic and lignin concentrations were measured at OD280 and OD325, respectively, and determined as mg·kg^−1^ FW [41].

### 4.7. Activities of Key Enzymes in Phenylpropanoid Metabolism

To extract key enzymes associated with phenylpropanoid metabolism in fresh-cut *G. elata*, the following procedure was employed: fresh-cut tissues (2 g) were finely ground and mixed with distinct extraction solutions. For the PAL, an extraction solution was prepared using a borate buffer (0.1 mol L^−1^) adjusted to pH 8.7. This buffer contained 2 mmol L^−1^ of ethylene diamine tetraacetic acid, 5 mmol L^−1^ of β-mercaptoethanol, and 40 g L^−1^ of polyvinylpyrrolidone. To isolate C4H, the extraction solution comprised Tris-HCl buffer (50 mmol L^−1^) at pH 8.9, supplemented with 15 mmol L^−1^ of β-mercaptoethanol, 5 mmol L^−1^ of AsA, 4 mmol L^−1^ of MgCl_2_, 1 mmol L^−1^ of phenylmethanesulfonyl fluoride, and 10 μmol L^−1^ of leupeptin. For 4CL, the extraction solution consisted of Tris-HCl buffer (50 mmol L^−1^) at pH 8.0, containing 0.1 mol L^−1^ of DTT and 25% (*v*/*v*) glycerol. The activities of PAL, C4H, and 4CL were assessed using the methodology outlined by Li et al. and were expressed as 10^6^ units per kg of protein (10^6^ U kg^−1^ protein) [16].

### 4.8. Microbiological Analysis

Microbiological analysis was conducted to determine the microbial colony counts in *G. elata* using SimPlate™ for HPC kits from IDEXX Laboratories (Westbrook, ME, USA), following the manufacturer’s guidelines. To prepare the sample, 1 g of *G. elata* was homogenized with 10 mL of sterile water using a previously disinfected pestle and mortar. The resulting homogenate was then transferred to a sterilized 50 mL centrifuge tube, and the volume was adjusted to 30 mL with sterile water. After centrifugation at 5000× *g* for 5 min, the supernatant was diluted 100-fold with sterile water for microbial analysis.

For the microbial analysis, one package of SimPlate Media was dissolved in 100 mL of sterile water, and 9.9 mL of the dissolved media was thoroughly mixed with 0.1 mL of the sample. This mixture was carefully poured onto a SimPlate plate that contained 100 holes, each with a 5 mm diameter. The plate was gently swirled to ensure that the mixture entered each hole evenly. After 12 h of incubation at 35 °C, the fluorescence emitted from the holes was recorded under ultraviolet light at 365 nm. The microbial colony counts were determined by converting the number of holes displaying fluorescence into Most Probable Number (MPN) using the MPN table provided by the manufacturer. The results were expressed as colony forming units per gram (CFU g^−1^).

### 4.9. Determination of Gene Expression

Gene expression was determined via quantitative real-time PCR (qRT-PCR) analysis. An RNeasy Plant Mini Kit (Takara, Japan) was used to extract RNA from tissue (1.0 g) in strict adherence to the exact procedure. RNA was purified using the manufacturer’s instructions of TRIzol^TM^ Plus RNA Purification Kit (Invitrogen™, Carlsbad, CA, USA) to remove DNA contamination. Synthesis of first-strand cDNA was performed using the Prime-Script^TM^ RT-PCR kit (TaKaRa, Dalian, China). qRT-PCR was performed using the KAPA 1-step qRT-PCR Kit (Bio-rad, Hercules, CA, USA) via CFX384 Touch Real-Time PCR Detection System. The *ACTIN* gene was used for quantitative normalization. Primers are shown in Table S1.

### 4.10. Data Analysis

All physiological data were examined in triplicate, and gene expression data were replicated in quadruplicate, with results shown as mean ± SE. These data were subjected to one-way analysis of variance (ANOVA) using SPSS version 17.0, and differences of *p* < 0.05 were considered significant.

## 5. Conclusions

**Figure 8:**
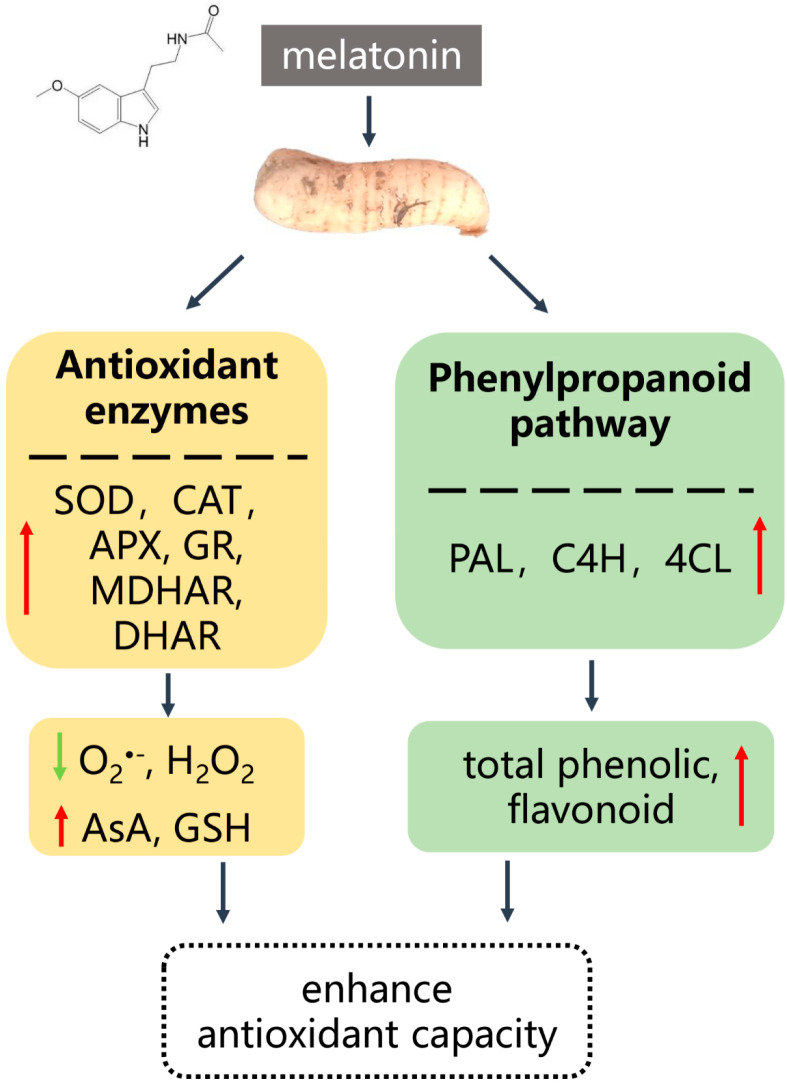
A speculation mechanism to explain the role of melatonin in maintaining quality in fresh-cut *G. elata.* Green arrows indicate decreased content, red arrows indicate increased content or up-regulated gene expression.

In conclusion, the application of MT to fresh-cut *G. elata* at 4 °C yields significant quality improvements. MT treatment effectively reduces the respiratory rate and MDA content, retards the decline in TA and TSS, and inhibits microbial growth, thereby enhancing product safety. Moreover, MT treatment enhanced the activity of antioxidant enzymes and effectively inhibited the activities of PPO and POD, thereby maintaining the postharvest quality of fresh-cut *G. elata*. Furthermore, MT treatment activates the phenylpropanoid pathway, increasing the content of total phenolics and flavonoids, enhancing the antioxidant capacity of fresh-cut *G. elata*. Figure 8 illustrates the potential mechanisms that MT enhances, contributing to the maintenance of the quality of fresh-cut *G. elata* by increasing antioxidant capacity and regulating phenylpropane metabolism.

## Data Availability

No new data were created or analyzed in this study. Data sharing is not applicable to this article.

## Supplementary Materials

The following supporting information can be downloaded at: https://www.mdpi.com/article/10.3390/ijms241814284/s1.
