# Supplementary material for: Melatonin Treatment Maintains the Quality of Fresh-Cut Gastrodia elata under Low-Temperature Conditions by Regulating Reactive Oxygen Species Metabolism and Phenylpropanoid Pathway
Source: Int J Mol Sci. 2023 Sep 19;24(18):14284. doi: 10.3390/ijms241814284 (PMC10531901; doi:10.3390/ijms241814284)
Supplement: Supplementary file 1 [file ijms-24-14284-s001.zip › ijms-2597246-supplementary.pdf]

Melatonin Treatment Maintains the Quality of Fresh-Cut *Gastrodia elata* under Low-Temperature Conditions by Regulating Reactive Oxygen Species Metabolism and Phenylpropanoid Pathway

Boyu Dong [1,2,*], Fangfang Da [1,2], Yulong Chen [1,2] and Xiaochun Ding [3,*]

Supplementary material

Table S1 Primer sequences used for real-time PCR.

GENE	PRIMER SEQUENCE
GeSOD	Forward: GAGGGCTTTGTTGAAGTTTGTGATG
	Reverse: CCTTCCTGATTTGCCATACGACTAC
GeCAT	Forward: CTTGCCTTCTGCCCGTCAATC
	Reverse: GCATTTGGAGGGAGCATGAGATAG
GePOD	Forward: GCTGTGATGCCTCAATTATGCTTG
	Reverse: GCCTCTAGCTGTGTCTTGATTCTG
GePPO	Forward: GCCAACATCGACCGCATCTG
	Reverse: TCTCTGACTCTGACCCTAACTAGC
GeAPX	Forward: CGTAAGGGCGGAAAACTGGATC
	Reverse: GAGCTGCTGAAAGGAGAATCGG
GeGR	Forward: TTGAAGCTAACAGAGTTGAGGTGAC
	Reverse: ACGGCTCCCAGTTGCAATTAAG
GeMDHAR	Forward: ACCTGCCGTACTTCTACTCTCG
	Reverse: GACCACCCTCCCATCCTTGAC
GeDHAR	Forward: ACCACTCTGGTTTGTTCGCA
	Reverse: ACTGAGCAACAACACTCCGT
GePAL	Forward: CCCAGGTCTCGTGCGTCGTTCT
	Reverse: TTTTACCTCTTGCCCGTGCGTT
GeC4H	Forward: CAGAGCCAGACACGCACAAGC
	Reverse: TAGGGAGACGACCACGGAGTG
Ge4CL	Forward: CCTCGGCTGCTGACTGACTG
	Reverse: GGTTGCGTTGGTCTCGCCTC
GeActin	Forward: GCGACAATGGAACTGGAATGG
	Reverse: TCGCCAGAATCCAGCACAAT
